# Beneficial Effects of *Spirulina* Supplementation in the Management of Cardiovascular Diseases

**DOI:** 10.3390/nu16050642

**Published:** 2024-02-25

**Authors:** Valeria Prete, Angela Carmelita Abate, Paola Di Pietro, Massimiliano De Lucia, Carmine Vecchione, Albino Carrizzo

**Affiliations:** 1 Department of Medicine, Surgery and Dentistry “Scuola Medica Salernitana”, University of Salerno, 84081 Baronissi, Italy; vprete@unisa.it (V.P.); aabate@unisa.it (A.C.A.); pdipietro@unisa.it (P.D.P.); cvecchione@unisa.it (C.V.); 2 Vascular Physiopathology Unit, IRCCS Neuromed, 86077 Pozzilli, Italy; massidelucia.m@libero.it

**Keywords:** *Spirulina*, hypertension, type 2 diabetes, hyperlipidemia, cardiovascular and cerebrovascular diseases

## Abstract

In recent decades, as a result of rising mortality rates due to cardiovascular diseases (CVDs), there has been a growing urgency to find alternative approaches to conventional pharmaceutical treatment to prevent the onset of chronic diseases. *Arthrospira platensis*, commonly known as *Spirulina*, is a blue-green cyanobacterium, classified as a “superfood”, used worldwide as a nutraceutical food supplement due to its remarkable nutritional value, lack of toxicity, and therapeutic effects. Several scientific studies have evaluated the cardioprotective role of *Spirulina*. This article presents a comprehensive review of the therapeutic benefits of *Spirulina* in improving cardio- and cerebrovascular health. It focuses on the latest experimental and clinical findings to evaluate its antihypertensive, antidiabetic, and antihyperlipidemic properties. The objective is to highlight its potential in preventing and managing risk factors associated with cardiovascular disease (CVD).

## 1. Introduction

*Arthrospira platensis* is a photosynthetic, microscopic filamentous blue-green microalga classified as a cyanobacterium belonging to the Microcoleaceae family [1]. *A. maxima* and *A. platensis*, commonly known as “*Spirulina*”, are the two most studied species for their considerable nutritional and therapeutic properties. *Spirulina* inhabits tropical regions, particularly alkaline lakes with a pH 11 and a high concentration of carbonate and bicarbonate salts. Additionally, these algae can survive in extreme environments, such as the frozen lakes of Antarctica [2,3]. These microorganisms were first discovered in Lake Texcoco in Mexico. The Aztecs were among the first to incorporate this microalga into their diet, particularly in the creation of a blue-green cake known as “tecuitlatl”, as unearthed by the Spanish army during their conquest of Mexico [4]. Since ancient times, *Spirulina* has been utilized for its beneficial characteristics. Today, *Spirulina* is still used in a wide range of applications. In recent decades, it has garnered the classification of a “superfood” because of its copious protein content (60–70% by dry weight) as well as its abundance of carbohydrates, essential fatty acids, vitamins, minerals, and pigments like carotenes, chlorophyll a, and phycocyanin [5]. Because of its impressive nutritional value, is widely utilized in both the food and pharmaceutical fields. In the food industry, *Spirulina* is used as a nutraceutical food supplement, added to foods such as baked goods, beverages, dairy products, sports supplements, and baby food [6]. On the other hand, the pharmaceutical sector has produced tablets, dehydrated powders or encapsulated forms, which are marketed as “nutraceuticals” [7].

As a consequence of the considerable market demand for *Spirulina* products, *S. platensis* has been classified as generally recognized as safe (GRAS) by the Food and Drug Administration (FDA) [8]. Many research studies have demonstrated that *Spirulina* has therapeutic functions such as antioxidant, anti-inflammatory, hypolipidemic, antidiabetic, and brain-protective properties [9,10,11]. Remarkably, the abundant presence of natural pigments endows *Spirulina* with antioxidant potential, notably carotenoids and C-phycocyanin [12,13]. Several research investigations indicated that β-carotene, diadinoxanthin, diatoxanthin, and C-phycocyanin exhibited very high scavenging activity [14,15]. Thanks to its antioxidant properties, this microalga is considered beneficial in preventing cardiovascular diseases [11]. Today, CVDs are the main cause of death globally [16]. Therefore, drug therapies used today to prevent certain predisposing disorders such as diabetes, hypertension, and dyslipidemia display many benefits and, at the same time, some adverse effects. For this reason, the use of nutraceuticals, such as *Spirulina*, has been shown promising results as a support therapy for the maintenance of cardiovascular health and the reduction in cardiovascular risk [17,18]. This renewed focus on functional-food-based therapy is now seen as a new strategy to achieve a healthy generation. In the 21st century, it is essential to follow the ideologies established by Hippocrates (460–377 B.C.), who claimed “Let food be your medicine” [19].

Accordingly, this review aims to pick up the latest experimental and clinical findings on the potential therapeutic properties of *Spirulina* for the treatment of cardiovascular diseases (CVDs). The article is divided into three main sections, which discuss the antihypertensive, antidiabetic, and antihyperlipidemic effects of *Spirulina*, focusing on general characteristics, clinical trials, animal studies, and mechanisms of actions. Overall, the review concludes that *Spirulina* is a powerful therapeutic tool for the treatment of CVDs and can be beneficial for individuals with hypertension, diabetes, and hyperlipidemia.

### Chemical Composition of Spirulina

*Spirulina* is mainly known for its valuable nutritional composition. Notwithstanding, significant differences in the main macromolecular composition were observed among the different phyla of this cyanobacterium [20]. In general, this microalga contains carbohydrates, lipids, vitamins, minerals, and a high protein content [21]. The high protein content on a dry weight basis is approximately 60% [22]. Specifically, the main proteins present in *Spirulina* consist of phycocyanin, allophycocyanin, and phycoerythrin [21]. Its low lipid composition is characterized by polyunsaturated essential fatty acids (PUFAs) such as omega-3 eicosapentaenoic acid (EPA), docosahexaenoic acid (DHA) [23], omega-6 arachidonic (AA), and γ linolenic acid (GLA) [24]. Moreover, *Spirulina* is also an excellent resource of minerals and vitamins. Researchers reported that the mineral composition includes potassium, calcium, magnesium, selenium, iron, zinc, and many others [25]. *Spirulina* is also valued for its content of vitamins, particularly B12 vitamin [26]. The vitamin C content is low due to exposure to light and heat during production, which can cause vitamin C degradation [25]. Finally, bioactive pigments including carotenoids, such as astaxanthin, zeaxanthin, and β-carotene [24] as well as chlorophylls [27], are present in *Spirulina*. Nevertheless, the macromolecular composition of different *Spirulina* strains can be strongly influenced by environmental and cultivation conditions, such as temperature, light, salinity, etc. [28]. For instance, a nitrogen and phosphorous limitation in *S. platensis* cultures can alter the biomass composition, leading to an increase in carbohydrate and lipid content [29]. Overall, its nutrient-rich composition had led to *Spirulina* being considered as a superfood that provides many health benefits.

## 2. Hypertension and Stroke

According to the World Health Organization (WHO), hypertension is considered one of the risk factors for CVDs and cerebrovascular diseases, such as cerebral hemorrhage and ischemic stroke, which are the leading cause of death worldwide [30,31]. The underlying causes of hypertension can be multiple and often unknown [32], resulting in increased resistance of peripheral vessels leading to increased blood pressure. As a result, systemic vascular damage occurs, and an adequate blood supply to the brain is not ensured [33,34]. The International Society of Hypertension Global Hypertension Practice Guidelines indicate that hypertension can be diagnosed as a systolic blood pressure (SBP) ≥ 140 mmHg and/or diastolic blood pressure (DBP) ≥ 90 mmHg [35]. Despite advances in the understanding and treatment of hypertension, the ability to effectively regulate blood pressure is still insufficient for many patients. This is complicated by the fact that high blood pressure may show no symptoms even for many years, thus posing a significant challenge for its management [36]. Moreover, it has been demonstrated that monotherapy is often not sufficient to lower blood pressure in hypertensive patients [37]. Egan and colleagues reported that administering combination therapy, which involves the use of multiple antihypertensives, could be an alternative approach to managing hypertension because it induces better cardiovascular outcomes than monotherapy [38]. Therefore, considering the capability of *Spirulina* supplementation to reduce blood pressure levels, it can be used to complement common antihypertensive therapy without replacing it.

### 2.1. Clinical Studies

The impact of the intake of *Spirulina* on blood pressure has been evaluated in several randomized clinical trials. Recently, a randomized, triple-blind, placebo-controlled trial was conducted on 48 hypertensive patients to assess the effects of consuming a salad dressing enriched with 2 g/day of *Spirulina* powder over 8 weeks in patients with hypertension compared to the placebo group. The results showed that the oral consumption of *Spirulina* dressing had a significant impact on the reduction in systolic (*p* = 0.02) and diastolic (*p* = 0.01) blood pressure [39]. In a similar study conducted by Mickze et al. [40], the authors confirmed the hypotensive effect of *Spirulina* further. They concluded that after four capsules of 0.5 g *Spirulina maxima* each or placebo administration in 40 patients with hypertension, a noteworthy reduction in systolic blood pressure (*p* = 0.0023) and stiffness index (*p* < 0.001) was observed from the baseline to three months. *Spirulina maxima* contained 60–70% protein, gamma-linolenic acid (GLA), beta carotene, iron and phycocyanin (PC).

In line with these findings, Martínez-Sámano and colleagues [41] showed that the administration of 4.5 g/day of *Spirulina maxima* for 12 weeks in 16 patients with systemic arterial hypertension (SAH), treated with angiotensin-converting enzyme (ACE) inhibitors, resulted in a significant reduction in systolic blood pressure (*p* < 0.05) at the end of treatment, while significant differences in diastolic blood pressure were not observed [41]. Moreover, a reduction in blood pressure was also highlighted in 36 Mexican patients with pre-hypertension (120–139 mmHg for SBP and 80–89 mmHg for DBP), hypertension stage 1 (140–159 mmHg for SBP and 90–99 mmHg for DBP), and hypertension stage 2 (higher values than 160 mmHg for SBP and 100 mmHg DBP) after the consumption of three tablets of 0.5 g *Spirulina* each every 8 h for six weeks (*p* < 0.001). Following *Spirulina* supplementation, the authors observed that 36% of patients achieved normal blood pressure, while patients with 1 or 2 hypertension stages decreased their levels to pre-hypertension levels (50%). Notably, younger patients showed greater responsiveness to *Spirulina*-induced blood-pressure-lowering than the other groups [42]. In contrast, a clinical investigation did not observe any significant effects of dietary supplementation with *Spirulina* on the blood pressure of healthy subjects compared to the placebo group. Subjects enrolled in this study consumed four capsules of 4.8 g *Spirulina* or a placebo after breakfast, lunch, and dinner for 17 days. The researchers did not observe statistically significant differences because the subjects involved in the study were healthy. In this case, it might be worth considering increasing the duration of treatment [43]. The parameters at baseline and after *Spirulina* supplementation of the included studies are shown in Table 1.

Unfortunately, there are a few clinical trials from the literature that evaluate the effect of *Spirulina* in hypertensive patients. Some of these studies lack a control group. Thus, further clinical trials with a larger number of patients treated with microalga and placebo are needed to evaluate its potential in reducing arterial blood pressure. In addition, not all studies reported specific details about the method of pressure measurement. It is not always discussed whether a manual or digital sphygmomanometer was used or if different measurements were taken. This lack of standardization makes it difficult to compare the results across studies.

### 2.2. Animal Studies

In the literature, studies have reported the significant antihypertensive effects of two tripeptides derived from the hydrolysis of blue algae *Spirulina platensis* powder, Ile-Gln-Pro (IQP) and Val-Glu-Pro (VEP), in vivo [44]. It was revealed that 8 weeks of oral treatment on spontaneously hypertensive rats (SHRs) led to a reduction in systolic and diastolic blood pressure, as well as a reduction in ventricular mass indices compared to the control group (*p* < 0.05). Compounds were administered 10 mg/kg every morning. Furthermore, the treatment had a significant impact on the expression levels of components of the renin–angiotensin system (RAS) in the myocardium [44].

Two other lyophilized peptides derived from *Spirulina* digestion were orally administered to SHR rats and have been shown to lower blood pressure for up to 8 h after supplementation compared to captopril, suggesting they have a longer-lasting ACE-inhibiting effect than captopril [45]. In another in vivo study conducted on SHR rats, tablets containing silicon-enriched *Spirulina* (15 mg *Spirulina* and 0.3 mg silicon) were administered orally for 3 months [46]. This supplementation induced a reduction in blood systolic (*p* < 0.001) and diastolic (*p* < 0.0001) pressure compared to SHR rats. In addition, they observed beneficial effects on vascular remodeling and improved vascular reactivity compared with *Spirulina* administration alone [46]. These findings suggest that the silicon enrichment contained in *Spirulina* may be able to improve arterial wall elasticity. Furthermore, *Spirulina* serves as a means by which elements can be assimilated in a form that the body can readily use. Consequently, it has been proven to be a promising dietary intervention for the treatment of hypertension in SHR rats.

Wang et al. showed that after ischemia/reperfusion, rats who were pre-treated with approximately 30 g of a *Spirulina*-enriched diet for four weeks showed reduced cerebral infarction and lower caspase-3 activity (*p* < 0.05) compared to the control group indicating a neuroprotective effect of blue-green alga [47]. The anti-apoptotic property was also demonstrated by Almeida and colleagues, who found that 30 days of *Spirulina* extract administration led to an increase in viable neurons in the perilesional fields of rats 24 h after an induced hemorrhage. All animals received 1.67 × 10^−2^ g of *Spirulina* extract diluted in 2 mL of distillated water via oral gavage [48]. Further studies are needed to investigate the potential of *Spirulina* as an agent to reduce injuries following cerebral hemorrhage and stroke.

### 2.3. Mechanisms of Action

The mechanisms of action of *Spirulina* in hypertension have been extensively studied. Several authors have proposed many mechanisms to demonstrate its efficiency in treating elevated blood pressure levels. According to Martínez-Sámano et al. [41], *Spirulina* supplementation induced a decrease in endothelial damage marker levels caused by an increase in blood pressure, such as sVCAM-1, sE-selectin, and endothelin-1, as well as the increase in glutathione peroxidase activity and oxidized glutathione levels, suggesting its potential to enhance endothelial function and its antioxidant properties that are beneficial in mitigating hypertension. Bioactive peptides derived from the hydrolysis of proteins contained in *Spirulina* have also been shown to possess antihypertensive properties. Only once released into the bloodstream can these peptides perform their function [49,50,51].

He et al. [52] demonstrated the total absorption of tripeptides through the intestinal epithelium using monolayers of human intestinal Caco-2 cells. They concluded that the main transport mechanism is the paracellular transport mechanism.

Pan et al. [44] assessed that two *Spirulina*-derived tripeptides can modulate the renin–angiotensin system (RAS), which has a central function in the regulation of blood pressure, specifically by activating the ACE2-Ang-(1-7)-Mas axis and inhibiting the ACE-Ang II-AT1 axis.

Recent work by our research group has demonstrated that a single decameric peptide called “SP6” (GIVAGDVTPI), derived from the gastrointestinal digestion (GID) of *Spirulina*, can lead to dose-dependent vasorelaxation in ex vivo mice vessels. In addition, this peptide can be effective as a blood-pressure-reducing agent by increasing PI3K/Akt/eNOS signaling, leading to the release of nitric oxide (NO), a well-known vasodilatation metabolite, whose bioavailability and signaling pathway are impaired in individuals with hypertension [53].

In line with our study, Majewski et al. [54] concluded that twelve peptides contained in *Spirulina* aqueous extract (SAE) can improve vascular function in the aorta of aged rats due to NO release and a decrease in superoxide production, associated with increased levels of p-eNOS and heme oxygenase-1 (HO-1), respectively [54].

Interestingly, another study proposed that a heptameric peptide (Thr-Met-Glu-Pro-Gly-Lys-Pro) can act as a mixed non-competitive inhibitor of ACE in human endothelial cells. Additionally, the expression of inducible NO synthase (iNOS) and endothelin-1 (ET-1) was reduced [55].

These results provide evidence to support the use of *Spirulina* as a preventive protection against vascular dysfunction and lay the foundation for its therapeutic use in hypotensive therapies in cardiovascular disease.

## 3. Diabetes

In the last decade, there has been a gradual increase in individuals afflicted with type 2 diabetes [56]. Diabetes mellitus (DM) represents a complex disease marked by elevated glucose levels and an increased basal metabolic rate because of a defect in insulin signaling.

In these conditions, hyperglycemia adversely affects the integrity of cellular membranes, leading to insulin resistance in both the liver and peripheral tissues and producing reactive oxygen species (ROS) [57].

Moreover, type 2 diabetes accounts for approximately 90% of all diagnosed cases of diabetes and is considered a risk factor for the development of CVDs, including myocardial infarction, peripheral vascular disease (PVD), heart failure, stroke, retinopathy, and neuropathy, as a result of microvascular and macrovascular complications due to hyperglycemia [58].

One of the proposed pharmacological approaches to treat hyperglycemia in diabetes is the utilization of metformin [59]. Generally, metformin has no impact on lipid profiles in subjects diagnosed with type 2 diabetes [60].

However, it is important to note that before considering the use of metformin, which can induce side effects of similar to those of digestive disorders, such as diarrhea and nausea [61], there are preventive measures that can be taken to avoid the development of diabetes. Pre-diabetes is a condition that occurs before the onset of diabetes, where blood sugar levels are higher than the normal range but not high enough to be considered type 2 diabetes.

Although it is often not possible to avoid the onset of the disease, exercise and food supplements can delay or improve the management of the disease because of improvements insulin sensitivity [62]. Furthermore, the bioactive ingredients contained in some food supplements, such as polyphenols, polysaccharides, and others, can affect the modulation of glucose metabolism [63].

Considerable research has focused on studying natural compounds with antihyperglycemic properties, such as *Spirulina*. Furthermore, unlike metformin, *S. platensis* not only reduces circulating glucose levels but can also influence lipoprotein metabolism, high levels of which are associated with diabetes. This would represent a possible cardiovascular benefit in diabetic patients [64].

*Spirulina* has gained attention as a functional food due to its potential to lower blood glucose levels, control cholesterol, and provide antioxidant benefits.

### 3.1. Clinical Studies

A recent randomized, double-blind, placebo-controlled study included 60 patients under usual treatment with metformin for type 2 diabetes [65]. The results suggest that 2 g of *Spirulina platensis* given as four capsules before meals, in addition to metformin therapy, markedly improved glycemic parameters, including glycosylated hemoglobin (HbA1c) (*p <* 0.001) and fasting blood glucose levels (FBS) (*p <* 0.001), compared to the placebo group under metformin treatment only. Hence, the supplementation of 2 g/day of *S. platensis* for 3 months is considered safe and free of side effects, making it an effective treatment option for the management of type 2 diabetes and its associated complications [65]. In line with this evidence, Alam et al. [66] evaluated that the administration of 7 g twice daily of *Spirulina* powder in patients with no pharmacological treatment can reduce FBS (*p* < 0.01) and postprandial glycemia (PPBS) similarly to the control group who received two capsules of 500 mg metformin before meals for 45 days. The authors observed no statistically significant differences in HbA1C levels induced by *Spirulina* treatment. As stated by the authors, this scenario could probably be due to the short duration of treatment and the small sample size. Further studies are needed to evaluate its pharmacological effect and its usefulness as a reliable alternative to classic antidiabetic agents [66]. Sowjanya and colleagues [67] divided diabetic patients into two groups. The first group (EG-1) was given 2 g of *Spirulina* contained in two snack bars (25 g each) in the mid-morning and evening. The second group (EG-2) was given two *Spirulina* capsules in the morning and evening, while the control group was given no supplementation. In both the EG-1 and EG-2 groups, their FBS, PPBS and HbA1c levels were significantly more reduced from the baseline to the endpoint both in males (*p* < 0.01) than in females (*p* < 0.05). In the EG-1 group, the reduction in FBS and PPBS levels was greater than in the EG-2 group, possibly due to the synergistic effects of other nutritional ingredients. In addition, the reduction in FBS and PPBS in females is lower than that in males [67]. Another clinical study showed that 2 g/day of *Spirulina* capsules, taken during lunch and dinner for 2 months, led to a reduction in FBS, PPBS, and HbA1c (*p* < 0.05) from the baseline to the endpoint [68]. A similar effect was induced by a dosage of 8 g of *Spirulina* administered daily for 3 months in tablet form to 50 patients with type 2 diabetes treated with their usual antidiabetic therapy, but the reduction in HbA1c levels was not significant. It is possible that *Spirulina* can lower serum glucose levels in the short term, but it may require a longer treatment period to affect hemoglobin A1C levels [69]. The reduction in the HbA1c level highlighted suggests an improvement in the regulation of long-term glucose management.

Moreover, in Cretan patients with non-alcoholic fatty liver disease (NAFLD), 6 g/day *Spirulina* (Greek production) supplementation for six months led to a significant reduction in the HOMA-IR index, a measure representing an enhancement in insulin sensitivity [70]. The main characteristics at the baseline and after *Spirulina* supplementation of the included studies are shown in Table 2.

Overall, *Spirulina* supplementation has been shown to be an effective agent for hyperglycemia; however, it is necessary to increase the number of studies and the duration of the treatment to confirm its effect on the regulation of HbA1c levels, indicative of long-term glucose management. These studies pave the way for future research on the use of nutraceuticals as an adjunct to basic therapy in managing diabetes mellitus.

### 3.2. Animal Studies

Similar research on the hypoglycemic property of *Spirulina* has been conducted in animal models. *Spirulina* oral supplementation in different concentrations (5, 10, and 15 mg/kg body weight) in streptozotocin-diabetic rats led to a decrease in FBS levels and, on the other hand, elevated plasma insulin and serum C-peptide concentrations (*p* < 0.05). Moreover, the researchers observed an increase in total hemoglobin levels in the *Spirulina*-treated group, which may be indirectly proportional to HbA1c formation, due to its ability to lower circulating glucose levels and its high iron content which is crucial for the metabolism of hemoglobin [71].

Further, the oral administration of *Spirulina* in albino diabetic rat models, at 10, 20, and 30 mg/kg body weight diluted in distillated water, resulted in a significant dose-dependent reduction in FBS compared to the diabetic control group (*p* < 0.01) [72].

Also, El-Sayed and colleagues [73] demonstrated that phenolic compounds and phycocyanin contained in *Spirulina* are responsible for the hypoglycemic effect. The four groups of diabetic rats treated for 30 days with oral *Spirulina* biomass suspension (50 mg/kg body weight), phycocyanin (50 mg/kg body weight), phycocyanobilin (982 µg/kg body weight), and phycopeptide (49 mg/kg body weight), respectively, showed a reduction in fasting blood glucose level and in the HOMA-IR score, which highlights insulin resistance, compared to the control and glibenclamide groups (*p* ≤ 0.05). In addition, a histopathological analysis revealed that diabetic rats treated with *Spirulina*, phycocyanin, and phycopeptide showed an improvement in their HOMA β-score which revealed an improvement in β cell function (*p* ≤ 0.05) [73].

### 3.3. Mechanism of Action

Although the mechanisms are not fully understood, *Spirulina* could be involved in pancreatic insulin secretion by islet β-cells or facilitate glucose transport from the blood to peripheral tissues [71].

The insulin-releasing impact of *S. platensis* occurs through a multitude of pathways, such as the adenylate cyclase/cAMP or phosphatidylinositol pathway or through direct influence on membrane depolarization [74].

Proteins extracted from *Spirulina* have been found to improve glucose entry into liver cells and promote glycogen synthesis by increasing the activities of hexokinase (HK) and pyruvate kinase (PK), which ultimately leads to lower blood glucose levels and improves insulin resistance.

Additionally, three peptides extracted from *Spirulina platensis* inhibit α-amylase, α-glucosidase, and dipeptidyl peptidase-4 (DPP-IV), which are critical enzymes involved in glycemic control. This makes them useful targets in treating type 2 diabetes [75].

The high fiber content of *Spirulina* may hinder glucose absorption, leading to a glucose-lowering effect [76].

According to Hozayen and colleagues [77], *Spirulina* could have a positive impact in diabetic rats enhancing serum adiponectin and decreasing TNF-α levels. High levels of adiponectin are commonly known to improve insulin sensitivity, while low levels of a pro-inflammatory cytokine TNF-α enhance glucose production in the liver and the ability of insulin to stimulate glucose uptake in peripheral tissues. Furthermore, the antioxidant capacity caused by *Spirulina* supplementation has been shown to increase GSH levels and SOD and GPx activity. As a result, it protects against oxidative-stress-induced cell damage associated with diabetes [77].

Some authors believe that the antioxidant capacity of *Spirulina* is attributed to phycocyanin. Selenium-bound phycocyanopeptide or/and phycocyanobilin are known for their antioxidant action in preventing diabetes-induced pancreatic cell damage, while chromium-bound phycocyanopeptide activates insulin receptors [73].

As a consequence of increased antioxidant enzymes, malondialdehyde (MDA) levels are decreased following *Spirulina* supplementation [78].

Lastly, Sadek et al. [79] provided evidence that *Spirulina* exerts its antidiabetic effect by attenuating the upregulation of gluconeogenic enzyme pyruvate carboxylase (PC) and pro-apoptotic Bax and caspase-3 (CASP-3) gene expression in diabetic rats, thereby resulting from its antioxidant activity. Furthermore, *Spirulina* has been shown to possess anti-apoptotic properties by mitigating the expression of pro-apoptotic MAPK pathways, consequently leading to the attenuation of apoptotic pathways induced by diabetes [79].

## 4. Hyperlipidemia

Hyperlipidemia refers to a condition characterized by elevated levels of lipids or lipoproteins in the blood that predisposes an individual to the development of atherosclerosis. Specifically, high blood levels of low-density lipoprotein cholesterol (LDL-C) and low blood levels of high-density lipoprotein (HDL) determine hypercholesterolemia. Blood cholesterol management guidelines state that LDL-C levels are one of the best predictors of CVDs [80]. In addition, the combination of other risk factors such as smoking and a sedentary lifestyle represents a significant correlation with atherosclerotic cardiovascular disorders [81]. Despite the high prevalence of hypercholesterolemia worldwide, the management of cardiovascular disease remains unsatisfactory. Typically, the conventional treatment of dyslipidemia involves the use of statins, which have been shown to reduce LDL-C levels and reduce CVD mortality. However, statin treatment is linked to adverse outcomes, including muscle weakness, muscle cramps, persistent myalgias, and elevations in creatine kinase levels [82]. Therefore, it is essential to enhance therapies by introducing adequate dietary therapy, including additional treatments, such as nutraceuticals, that can control plasma lipid and lipoprotein levels to improve outcomes for dyslipidemic individuals.

To this end, several studies have demonstrated *Spirulina’s* valuable effect in reducing plasma concentrations of LDL-C and triglycerides and increasing HDL-C levels following its supplementation, resulting in a reduced risk of developing cardiovascular diseases.

The effect of *Spirulina* on plasma lipid levels is independent of the dose administered and has no toxic effects [83]. Overall, *Spirulina* supplementation is shown to be effective in the treatment of hyperlipidemia and subsequent atherosclerosis to improve lipid profiles in patients.

### 4.1. Clinical Studies

Several recent clinical studies have been conducted to evaluate the lipid-lowering effect of *Spirulina* in obese subjects. Obesity is correlated with high blood lipid levels representing a risk factor linked to CVD.

Zeinalian and colleagues [84] conducted a study on obese individuals who were administrated 1 g (500 mg twice a day) of *Spirulina pills* for 3 months and observed a notable reduction in total cholesterol (TC) (*p* = 0.002). Furthermore, an increase in high-density lipoprotein-cholesterol (HDL-C) (*p* = 0.05) was observed with no significant change in triglycerides (TG) or low-density lipoprotein (LDL) between the baseline and after *Spirulina* treatment [84].

A further study in humans enrolled obese subjects, already undergoing antihypertensive treatment, who received four capsules of 0.5 g each of *Spirulina maxima*, taken daily in the morning, or a placebo for the same duration as the previous study. *Spirulina* supplementation resulted in a decrease in LDL-C (*p* < 0.001) and TC (*p* < 0.001) levels between the baseline and after administration, but it was not effective in reducing other lipid levels [85].

Although it is known that exercise can reduce CVD risk factors by improving the serum lipid profile, the synergistic action with *Spirulina capsules* has been evaluated in obese and sedentary individuals. A randomized double-blind study assessed the combined action of *Spirulina* supplementation (4.5 g/day for 6 weeks) in dyslipidemic subjects. Although synergetic action has proven advantageous, *Spirulina* supplementation alone has been shown to lower total cholesterol, LDL-C, and triglycerides, and to increase HDL-C in 6 weeks compared to control group [86].

In contrast, another clinical study evaluated the effect of *Spirulina* supplementation and high-intensity interval training on 20 overweight or obese women for 4 weeks. Administration was taken orally in pill form, once in the morning and again 48 h after the last exercise session. There were no significant differences observed in serum lipid levels, probably because of the low number of cohort participants involved in the study. Another limitation is the participation of only women [87].

In a study by Karizi et al., the efficacy of blue-green algae in reducing lipid levels in type 2 diabetes mellitus (T2DM) was evaluated. The authors studied the simultaneous action of metformin therapy and 2 g/day *Spirulina* in diabetic patients for 12 weeks. The supplementation of *S. platensis* before meals has been shown to have a hypolipidemic action and to be a valuable adjunct to metformin therapy. At the end of the intervention period, the researchers found a significant decrease in plasma values of atherogenic lipids and an increase in HDL levels compared to the baseline (*p* < 0.001) [65]. In a Cretan population, 3 months of *Spirulina* administration lowered all non-high-density lipoprotein cholesterol levels (*p* < 0.001), but the HDL-C levels remained unchanged [88].

Other studies have examined the potential role of *Spirulina*-fortified dressing or sauce as a functional food. Far and colleagues [39] emphasized a significant reduction in triglycerides (*p* < 0.01) in hypertensive patients when given salad dressing that contained 2 g of *Spirulina* powder [39], while the results from the study by Mazloomi et al. [89] confirmed the potential benefits in NAFLD patients. The investigation suggests that sauce prepared with 2 g of *Spirulina* may improve NAFLD by reducing the grade of fatty liver and liver enzymes (ALT and AST) as well as TG reduction (*p* = 0.03) and HDL-C (*p* = 0.02) levels increase between baseline and the end of the treatment. Additionally, the atherogenic index was significantly decreased (*p* = 0.007) [89]. The influence of a liquid extract of Arthrospira called “Spirulysat^®^” was examined in individuals with metabolic syndrome, demonstrating a reduction in triglycerides and an increase in HDL levels at the end of supplementation compared to placebo group. *Spirulina* water extract contained phycocyanin, polysaccharides and proteins, amino acids, enzymes, vitamins, and mineral salts [90]. On the other hand, the intake of *Spirulina* supplements for 17 days, with four capsules taken after each principal meal induced no effect either on plasma lipid levels or markers for synthesis and intestinal cholesterol absorption [43]. The basic characteristics of all included studies are summarized in Table 3.

However, most studies have evaluated the antihyperlipidemic effects of *Spirulina* in overweight and obese subjects. Therefore, the impact of this microalga on individuals with different conditions remains unclear. For this reason, further research is still required to fully understand and confirm *Spirulina*’s benefits in reducing serum lipid concentrations in a heterogeneous population sample.

### 4.2. Animal Studies

The impact of *Spirulina* on plasma lipid concentrations in animal models has also been assessed in the literature. The investigation by El-Sayed et al. [73] reported that 30 days of *Spirulina* feeding and its extracts, such as phycocyanin (PHY), phycocyanopeptide, and phycocyanobilin, resulted in a significant decrease in atherogenic serum lipid levels and an increase in HDL-cholesterol levels in diabetic rats. Specifically, it was emphasized that the main effect on HDL and LDL concentrations was mainly attributable to the antioxidant activity of phycocyanin and phycocyanopeptide [73].

In diabetic rats, similar results were presented by Nasirian et al. [91] who also observed a decrease in malondialdehyde (MDA) levels (*p* < 0.05), an index of lipid peroxidation, compared to the control group. Moreover, increasing concentrations of *Spirulina* extracts diluted in water were found to be correlated with high levels of antioxidant liver enzymes when orally consumed (*p* < 0.05) [91]. Furthermore, *Spirulina* concentrated (SPC), PHY, and PHY residues were added in rats’ high-cholesterol diet for 5 days. PHY has been shown to significantly reduce liver cholesterol levels compared with the SPC diet [92]. An improvement in lipid profiles was observed in two groups of hypercholesterolemic male rabbits treated orally for four weeks with different doses of algal alkaloid extract (33 and 66 mg/kg) (*p* ≤ 0.05) [93]. The researchers discovered that a *Spirulina*-enriched soy yogurt diet for administered for 4 weeks and oral treatment with *Spirulina* powder (500 mg/kg) for 8 weeks in hypercholesterolemic mice provided a protective effect against hepatic steatosis by reducing hepatic fat accumulation [94]. These findings are similar to those of Li et al. [95], who linked hepatic and plasma hypolipidemic activity to the ability to regulate the gut microbiota. *Spirulina* polysaccharides (150 mg/kg/day) were administered intragastrically for 8 weeks, thereby manifesting lipid-reducing effects in rats with hypercholesterolemia compared to the high-fat diet group (*p* < 0.01) [95]. These findings indicate that *Spirulina* may positively impact lipid profiles, which could lead to improved cardiovascular health.

### 4.3. Mechanism of Action

Several studies have shown that *Spirulina* supplementation can significantly lower levels of LDL-C, TC, and TG while increasing levels of HDL-C. These beneficial effects on blood lipid profiles are attributed to its nutritional content, but its mechanisms of action are not fully understood.

In the literature, it has been documented that *S. platensis* could impact lipid metabolism by down-regulating lipogenesis-related genes such as transcription factor-1c (SREBP-1c), acetyl CoA carboxylase (ACC), and peroxisome proliferator-activated receptor-g (PPARγ). Furthermore, researchers suggested an increase in peroxisome proliferator-activated receptor-a (PPARα) and adenosine 5′-monophosphate-activated protein kinase (AMPK) gene expression levels, which are involved in the regulation of fatty acid oxidation [95,96]. Related to the low levels of SREBP-1c, some authors have shown an increase in PGC-1α levels, a cofactor implicated both in the regulation of lipid oxidation gene expression and in hepatic mitochondrial biogenesis through the PGC-1α/Tfam/mtDNA pathway in the liver [97]. Additionally, *Spirulina* inhibits HMG COA reductase activity, a key enzyme in cholesterol synthesis, and increases the activity of Lecithin cholesterol acyltransferase (LCAT), which plays a central role in the reverse cholesterol transport process [98]. One of the first studies conducted on hypercholesterolemic Wistar rats revealed an improvement in lipoprotein lipase (LPL) and hepatic triglyceride lipase (H-TGL) activity [99]. Consequently, these alterations resulted in reduced levels of plasma cholesterol and triglycerides and elevated levels of HDL-C. Several investigations have proposed that *Spirulina* can positively impact dysbiosis by altering the composition of gut microbiota, which positively correlates with blood lipid levels [95,96].

According to Oriquat, *Spirulina* could alleviate non-alcoholic fatty liver disease (NAFLD) through the modulation of the hepatic expression of miR-122, miR-34a, and miR-21, as well as the SREBP-1c, SIRT1, and HPB1 genes [100].

Many researchers have attributed the mechanism underlying the hypolipidemic effect to C-phycocyanin, a blue-green pigment contained in *Spirulina*.

A study by Nagaoka et al. revealed, for the first time, that the consumption of C-phycocyanin (PHY) could suppress the intestinal absorption of cholesterol, as C-phycocyanin can bind to bile acids in the jejunum, resulting in an effect on the higher fecal excretion of cholesterol, which ultimately leads to a reduction in serum cholesterol levels [92]. Furthermore, according to Han et al., C-phycocyanin and glycolipid H-b2 inhibit pancreatic lipase activity in a dose-dependent manner [101]. A recent study also suggested that PHY could improve liver fat accumulation by regulating AMPK pathway [102]. PHY has antioxidant action, anti-inflammatory and free radical scavenging [13,15], thus exerting an inhibitory influence on lipid peroxidation. This confirmed the findings of Riss and colleagues which demonstrated that PHY can reduce the aortic fatty streak area by modulating NADPH oxidase and reducing superoxide anion accumulation, a marker of early atherosclerosis [103]. Phycocyanobilin (PCB), its tetrapyrrole chromophore, also showed an antioxidant effect, significantly increasing HMOX1 expression in aortic atherosclerotic plaques of ApoE-deficient mice, thus inducing atheroprotection [104]. Although several mechanisms by which *Spirulina* improves hyperlipidemia and consequently atherosclerosis have been suggested in the literature, further studies are needed to further investigate the mechanisms involved.

## 5. Conclusions

The present review was conducted to analyze the efficacy of *Spirulina platensis* supplementation on the current experimental and clinical findings in hypertension, diabetes, and dyslipidemic conditions.

According to the latest clinical research, consuming *Spirulina* has no health risks, as it has been classified as generally recognized as safe (GRAS) by the Food and Drug Administration (FDA). Moreover, considering its “natural” origins, consumers prefer its use for human health promotion over pharmacological treatment. This classification has led researchers to investigate its possible beneficial role in different cardiovascular pathologies.

**Figure 1:**
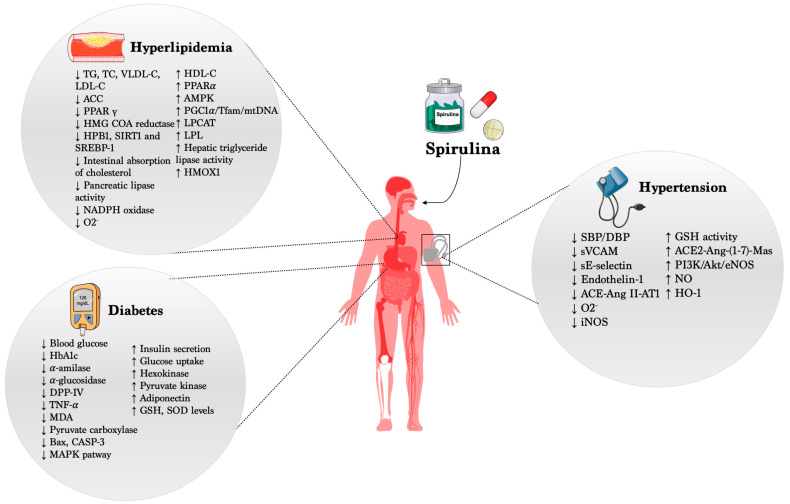
Beneficial effects of *Spirulina* in CVDs.

Several reports have demonstrated and highlighted the beneficial action of *Spirulina* in different cardio- and cerebrovascular diseases, acting by preventing or, at least, limiting cardiovascular risk factors such as high blood pressure, hyperglycemia, and hyperlipidemia (Figure 1). On the other hand, it is important to point out that its administration in healthy subjects did not evoke any modification of the physiological parameters, fully supporting the use of *Spirulina* as a potential preventive compound able to counteract the onset and progression of CVDs.

Despite the scientific literature highlighting the potential beneficial role of *Spirulina*, it is important to emphasize that future research testing its therapeutic effects in a large heterogeneous population is needed, also taking into account the ethnicity, lifestyle, behavior, and gender-specific actions that can influence the physiological responses to nutraceutical treatment.

Therefore, the first goal to be achieved is to expand the number of studies evaluating the effects of *Spirulina* on populations from different regions of the world, so that the beneficial effects observed to date can be assessed unequivocally.

Environmental and growth conditions in which the algae are cultivated can affect the nutritional and pharmacological-like properties of the final *Spirulina* compound. Thus, the second milestone to achieve is to fully characterize the macronutrient composition of different strains type of *Spirulina*, aiming to establish a common denominator on macronutrients that can evoke the greatest beneficial effect on human health.

Finally, two big questions lie in the treatment dosage and timing to appreciate the impact of *Spirulina* treatment in specific vascular diseases. From the antihypertensive effect to the antidiabetic and antihyperlipidemic effects, the dosage ranges from 1 to 8 mg/die, and on the same line, the period of treatment changes from a minimum of 17 days to 12 weeks intra-pathology. Thus, until now, it has not been possible to establish a selective guideline on “how to administer” *Spirulina* for the prevention or treatment of specific cardiovascular diseases. Therefore, only by increasing the number of specific studies on CVDs will it be possible to achieve this final milestone in establishing the timing and dosages of *Spirulina* to be used, helping to define its preventive or adjuvant-drug-therapy use necessary to fight and contain the cardiovascular risk.

In conclusion, based on these data, more rigorous studies should be planned in the future aiming to address these critical questions, putting the foundations for developing a common guideline on “how and when” to use *Spirulina*.

## Figures and Tables

**Table 1:** Detailed characteristics of the included studies on hypertensive patients.

References	Patients’ Cohort	Dose of *Spirulina*	Duration Treatment (Weeks/Months)	Outcomes in *Spirulina* Group; (*p*-Value)	Outcomes in Control Group; (*p*-Value)	*p*-Value
				**SBP (mmHg)**	**SBP (mmHg)**	
				Baseline 144.72 ± 2.98	Baseline 140.59 ± 3.81	0.39
				End 138.46 ± 2.98	End 141.07 ± 3.22	0.55
**Ghaem Far et al., 2021** [39]	*Spirulina* group;	2 g/day	8 weeks	*p* = 0.02	*p* = 0.89	
	Placebo group			**DBP (mmHg)**	**DBP (mmHg)**	
				Baseline 96.60 ± 1.76	Baseline 90.94 ± 3.6	0.17
				End 92.58 ± 2.21	End 89.70 ± 2.98	0.43
				*p =* 0.03	*p* = 0.61	
				**SBP (mmHg)**	**SBP (mmHg)**	
				Baseline 149 ± 7	Baseline 150 ± 7	0.36
				End 143 ± 9	End 151 ± 9	<0.001
**Miczke et al., 2016** [40]	*Spirulina* group;	2 g/day	3 months	*p =* 0.0023	*p =* 0.38	
	Placebo group			**DBP (mmHg)**	**DBP (mmHg)**	
				Baseline 84 ± 9	Baseline 85 ± 9	0.81
				End 79 ± 9	End 86 ± 7	<0.001
				*p =* 0.057	*p =* 0.19	
				**SBP (mmHg)**	**SBP (mmHg)**	
				Baseline 140.38 ± 9.04	Baseline 140.75 ± 7.03	n.s
				End 126.50 ± 5.53	End 140± 6.05	*p* < 0.05
**Martínez-Sámano et al., 2018** [41]	*Spirulina* group;	4.5 g/day	12 weeks	*p <* 0.05	*n.s*	
	Placebo group			**DBP (mmHg)**	**DBP (mmHg)**	
				Baseline 83.75 ± 5.31	Baseline 84.25 ± 5.28	n.s
				End NA	End NA	n.s
				n.s	n.s	
				**SBP (mmHg)**		
				Baseline 120 ± 9		
				End 109 ± 9		
**Torres-Duran et al., 2007** [42]	*Spirulina* group	4.5 g/day	6 weeks	*p <* 0.001	NA	
				**DBP (mmHg)**		
				Baseline 85 ± 9		
				End 79 ± 8		
				*p <* 0.05		
				**SBP (mmHg)**	**SBP (mmHg)**	
				Baseline −	Baseline −	n.s
				End 113.9 ± 13.7	End 114.4 ± 14.5	n.s
**van den Driessche et al., 2020** [43]	*Spirulina* group;	4.8 g/day	17 days	n.s	n.s	
	Placebo group			**DBP (mmHg)**	**DBP (mmHg)**	
				Baseline −	Baseline −	n.s
				End 75.4 ± 9.4	End 74.9 ± 9.5	n.s
				n.s	n.s	

Abbreviations: SBP, systolic blood pressure; DBP, diastolic blood pressure; NA, not available; n.s, not significant.

**Table 2:** Detailed characteristics of the included studies on diabetic patients.

References	Patients’ Cohort	Dose of *Spirulina*	Duration Treatment (Weeks/Months)	Outcomes in *Spirulina* Group; (*p*-Value)	Outcomes in Control Group; (*p*-Value)	*p*-Value
				**HbA1c (mg/dL)**	**HbA1c (mg/dL)**	
				Baseline 8.87 ± 0.29	Baseline 8.47 ± 0.21	0.65
				End 7.44 ± 0.20	End 8.15 ± 0.17	NA
**Karizi et al.,** **2022** [65]	*Spirulina* + Metformin group;	2 g/day	3 months	*p* = 0.001	*p =* 0.016	
	Placebo + Metformin group			**FBS (mg/dL)**	**FBS (mg/dL)**	
				Baseline 167.30 ± 4.34	Baseline 227.60 ± 67.85	0.47
				End 136.33 ± 4.42	End 165.47 ± 3.37	NA
				*p =* 0.001	*p =* 0.99	
				**HbA1c (mg/dL)**	**HbA1c (mg/dL)**	
				Baseline 9.73 ± 1.92	Baseline 9.61 ± 1.49	0.862
				End 9.95 ± 2.11	End 9.15 ± 2.03	0.303
				*p =* 0.525	*p =* 0.459	
				**FBS (mg/dL)**	**FBS (mg/dL)**	
**Alam et al., 2016** [66]	*Spirulina* group;	7 g/day	45 days	Baseline 245.53 ± 78.95	Baseline 227.60 ± 67.85	0.525
	Placebo + Metformin group			End 204.87 ± 78.15	End 191.80 ± 78.91	0.65
				*p =* 0.003	*p =* 0.212	
				**PPBS (mg/dL)**	**PPBS (mg/dL)**	
				Baseline 345.73 ± 98.33	Baseline 329.60 ± 72.92	NA
				End 303.67 ± 96.16	End 282.80 ± 99.90	NA
				NA	NA	
				**HbA1c (mg/dL)**	**HbA1c (mg/dL)**	
				Baseline EG-I M 9.19 ± 0.88; W 8.88 ± 0.70 EG-II M 7.33 ± 0.54; W 7.20 ± 0.33	Baseline M 8.00 ± 1.05; W 8.64 ± 0.79	NA
				End EG-I M 7.11 ± 0.64; W 7.64 ± 0.48 EG-II M 6.48 ± 0.36; W 6.58 ± 0.35	End M 7.98 ± 1.03; W 8.62 ± 0.74	NA
				EG-I M *p* < 0.01; W *p* < 0.01 EG-II M *p* < 0.01 W *p* < 0.01	M n.s; W n.s	
	EG1 group;			**FBS (mg/dL)**	**FBS (mg/dL)**	
**Sowjanya et al., 2022** [67]	EG2 group	2 g/day	3 months	Baseline EG-I M 138.00 ± 18.39; W 128.08 ± 11.76 EG-II M 135.02 ± 18.22; W 132.33 ± 10.89	Baseline M 146.10 ± 25.29; W 135.12 ± 10.27	NA
	Control group			End EG-I M 122.21 ± 14.48; W 111.00 ± 14.48 EG-II M 119.31 ± 14.33; W 123.12 ± 9.81	End M 141.43 ± 20.84; W 130.12 ± 9.76	
				EG-I M *p* < 0.01; W *p* < 0.01 EG-II M *p* < 0.01; W *p* < 0.05	M n.s; W n.s	NA
				**PPBS (mg/dL)**	**PPBS (mg/dL)**	
				Baseline EG-I M 210.33 ± 28.99; W 212.12 ± 39.45 EG-II M 197.45 ± 23.31 W 190.03 ± 14.86	Baseline M 206.17 ± 22.83; W 179.24 ± 17.82	NA
				End EG-I M 165.56 ± 25.35; W 175.58 ± 32.11 EG-II M 171.28 ± 24.77 W 175.50 ± 18.38	End M 202.37 ± 22.76; W 172.09 ± 15.49	NA
				EG-I M *p* < 0.01; W *p* < 0.01 EG-II M *p* < 0.01; W *p* < 0.05	M n.s; W n.s	
				**HbA1c (mg/dL)**	**HbA1c (mg/dL)**	
				Baseline 9.0 ± 2.3	Baseline 8.7 ± 1.5	NA
				End 8.0 ± 1.3	End 8.7 ± 1.3	NA
				*p* < 0.05	n.s	
				**FBS (mg/dL)**	**FBS (mg/dL)**	
				Baseline 161.7 ± 48.6	Baseline 164.3 ± 59.4	NA
**Parikh et al.,** **2001** [68]	*Spirulina* group;	2 g/day	2 months	End 142.4 ± 27.4	End 165.1 ± 44.3	NA
	Control group			NA	NA	
				**PPBS (mg/dL)**	**PPBS (mg/dL)**	
				Baseline 264.9 6 65.2	Baseline 215.2 6 67.3	NA
				End 248.8 6 68.9	End 212.3 6 57.6	NA
				NA	NA	
				**FBS (mg/dL)**		
**Beihaghi et al., 2017** [69]	*Spirulina* group;	8 g/day	3 months	Baseline 158.1 ± 44.2	NA	
	Control group			End 127.8 ± 36.7		
				NA		

Abbreviations: HbA1c, glycosylated hemoglobin; FBS, fasting blood glucose levels; PPBS, post-prandial blood glucose; EG1, Experimental group-1 who received *Spirulina* snack bar; EG2, Experimental group-2 who received *Spirulina* capsules; NA, not available. n.s, not significant.

**Table 3:** Detailed characteristics of the included studies on hyperlipidemic patients.

References	Patients’ Cohort	Dose of *Spirulina*	Duration Treatment (Weeks/Months)	Outcomes in *Spirulina* Group; (*p*-Value)	Outcomes in Control Group; (*p*-Value)	*p*-Value
				**TG (mg/dL)**	**TG (mg/dL)**	
				Baseline 144.13 ± 57.57	Baseline 156.14 ± 79.01	0.486
				End 136.65 ± 60.80	End 140.88 ± 72.73	NA
				*p =* 0.365	*p* = 0.052	
				**LDL-C (mg/dL)**	**LDL-C (mg/dL)**	
				Baseline 116.27 ± 34.79	Baseline 119.90 ± 21.69	0.725
				End 115.42 ± 28.61	End 116.68 ± 21.31	NA
**Zeinalian et al., 2017** [84]	*Spirulina* group;	1 g/day	12 weeks	*p =* 0.886	*p =* 0.196	
	Control group			**HDL-C (mg/dL)**	**HDL-C (mg/dL)**	
				Baseline 36.55 ± 10.21	Baseline 34.88 ± 11.56	0.385
				End 38.75 ± 8.84	End 38.37 ± 9.44	NA
				*p* = 0.05	*p* = 0.001	
				**TC (mg/dL)**	**TC (mg/dL)**	
				Baseline 190.48 ± 35.25	Baseline 187.25 ± 27.10	0.123
				End 180.10 ± 31.13	End 183.03 ± 28.07	NA
				*p* = 0.002	*p* = 0.09	
				**TG (mmol/L)**	**TG (mmol/L)**	
				Baseline 1.9 ± 1.0	Baseline 2.0 ± 1.2	0.334
				End 1.8 ± 0.9	End 2.1 ± 1.1	0.224
				*p* = 0.633	*p* = 0.981	
				**LDL-C(mmol/L)**	**LDL-C (mmol/L)**	0.412
				Baseline 3.5 ± 0.9	Baseline 3.6 ±0.9	<0.001
				End 3.0 ± 0.6	End 3.6 ± 0.9	
**Szulinska et al., 2017** [85]	*Spirulina* group;	2 g/day	3 months	*p <* 0.001	*p* = 0.223	
	Placebo group			**HDL-C (mmol/L)**	**HDL-C (mmol/L)**	
				Baseline 1.4 ± 0.3	Baseline 1.3 ± 0.4	0.357
				End 1.4 ± 0.3	End 1.2 ± 0.3	0.002
				*p* = 0.227	*p* = 0.204	
				**TC (mmol/L)**	**TC (mmol/L)**	
				Baseline 5.5 ± 1.1	Baseline 5.2± 0.9	0.191
				End 5.2± 0.9	End 5.4± 0.8	0.150
				*p <* 0.001	*p* = 0.306	
				**TG (mg/dL)**	**TG (mg/dL)**	
				Baseline *Sp* 167 ± 11 *Sp* + exercise 184 ± 40	Baseline Placebo 160 ± 6. Placebo + exercise 180 ± 25	NA
				End *Sp* 148 ± 19 *Sp* + exercise 156 ± 29	End Placebo 153 ± 12 Placebo + exercise 164 ± 21	NA
	*Spirulina* group;			*Sp p <* 0.05 *Sp* + exercise *p <* 0.05	Placebo *p =* 0.156 Placebo + exercise *p =* 0.096	
	*Spirulina* + exercise group;			**LDL-C (mg/dL)**	**LDL-C (mg/dL)**	
**Hernández-Lepe** **et al., 2019** [86]	Control group;	4.5 g/day	6 weeks	Baseline *Sp* 148 ± 33 *Sp* + exercise 141 ± 29	Baseline Placebo 140 ± 29 Placebo + exercise 148 ± 33	NA
	Control + exercise group			End *Sp* 128 ± 32 *Sp* + exercise 101 ± 34	End Placebo 135 ± 27 Placebo + exercise 124 ± 33	NA
				*Sp p = 0.060* *Sp* + exercise *p* < 0.05	Placebo *p =* 0.650 Placebo + exercise *p* < 0.05	
				**HDL-C (mg/dL)**	**HDL-C (mg/dL)**	
				Baseline *Sp* 29 ± 6 *Sp* + exercise 30 ± 6	Baseline Placebo 28 ± 8 Placebo + exercise 28 ± 6	NA
				End *Sp* 35 ± 10 *Sp* + exercise 40 ± 10	End Placebo 31 ± 5 Placebo + exercise 33 ± 6	NA
				*Sp p* < 0.05 *Sp* + exercise *p* < 0.05	Placebo *p* = 0.172 Placebo + exercise *p* < 0.05	
				**TC (mg/dL)**	**TC (mg/dL)**	
				Baseline *Sp* 233 ± 21 *Sp* + exercise 226 ± 22	Baseline Placebo 219 ± 16 Placebo + exercise 232 ± 23	NA
				End *Sp* 212 ± 23 *Sp* + exercise 189 ± 20	End Placebo 213 ± 18 Placebo + exercise 208 ± 28	NA
				*Sp p* < 0.05 *Sp* + exercise *p* < 0.05	Placebo *p* = 0.412 Placebo + exercise *p* < 0.05	
				**TG (mg/dL)**	**TG (mg/dL)**	
				Baseline 94.09 ± 61.44	Baseline 86.57 ± 33.26	NA
				End 93.82 ± 55.29	End 74.76 ± 18.41	NA
	HIIT+ *Spirulina* group;			*p* = 0.97	*p* = 0.22	
**Golestani et al.,** **2021** [87]	HIIT+ Placebo group	1 g/day	4 weeks	**LDL-C (mg/dL)**	**LDL-C (mg/dL)**	
				Baseline 132.43 ± 19.80	Baseline 130.93 ± 17.23	NA
				End 125.32 ± 30.59	End 123.04 ± 10.90	NA
				*p* = 0.37	*p* = 0.21	
				**HDL-C (mg/dL)**	**HDL-C (mg/dL)**	
				Baseline 54.23 ± 6.97	Baseline 59.49 ± 3.85	
				End 57.62 ± 5.18	End 60.37 ± 5.39	
				*p* = 0.15	*p* = 0.65	
				**TC (mg/dL)**	**TC (mg/dL)**	
				Baseline 167.35 ± 39.48	Baseline 170.81 ± 26.62	NA
				End 163.24 ± 38.85	End 159.13 ± 23.85	NA
				*p* = 0.69	*p* = 0.14	
				**TG (mg/dL)**	**TG (mg/dL)**	
				Baseline 184 ± 12.9	Baseline 181 ± 11.7	0.96
				End 113.63 ± 40.56	End 180.37 ± 63.76	NA
				*p* = 0.001	*p* = 0.001	
				**LDL-C (mg/dL)**	**LDL-C (mg/dL)**	
				Baseline 112 ± 9.12	Baseline 110 ± 8.6	0.87
				End 73.47 ± 37.4	End 110.97 ± 47.54	NA
**Karizi et al.,** **2022** [65]	*Spirulina* + Metformin group;	2 g/day	3 months	*p* = 0.001	*p* = 0.001	
	Placebo + Metformin group			**HDL-C (mg/dL)**	**HDL-C (mg/dL)**	
				Baseline 42 ± 1.3	Baseline 42 ± 1.3	0.78
				End 45.1 ± 6.83	End 40.5 ± 6.25	NA
				*p* = 0.001	*p* = 0.001	
				**TC (mg/dL)**	**TC (mg/dl)**	
				Baseline 190 ± 8.8	Baseline 186 ± 8.0	0.78
				End 149.07 ± 40.02	End 187.63 ± 43.39	NA
				*p* = 0.001	*p* = 0.001	
				**TG (mg/dL)**		
				Baseline 184.8 ± 16.8		
				End 138.9 ± 23.8		
				*p <* 0.0001		
				**LDL-C (mg/dL)**		
				Baseline 200.6 ± 27.6		
**Mazokopakis et al., 2014** [88]	*Spirulina* group	1 g/day	3 months	End 183 ± 23.6	NA	
				*p <* 0.0001		
				**HDL-C (mg/dL)**		
				Baseline 38 ± 6.9		
				End 39.6 ± 6.3		
				*p =* 0.0002		
				**TC (mg/dL)**		
				Baseline 275.5 ± 29.9		
				End 250.3 ± 26.7		
				*p <* 0.0001		
				**TG (mg/dL)**	**TG (mg/dL)**	
				Baseline 195.77 ± 16.74	Baseline 161.21 ± 15.26	0.07
				End 175.13 ± 16.46	End 155.42 ± 16.51	0.29
				*p =* 0.01	*p =* 0.44	
				**LDL-C (mg/dL)**	**LDL-C (mg/dL)**	
				Baseline 108.22 ± 5.62	Baseline 108.57 ± 5.61	0.96
				End 106.13 ± 5.66	End 113.63 ± 5.46	0.35
**Ghaem Far et al., 2021** [39]	*Spirulina* group;	2 g/day	8 weeks	*p =* 0.55	*p =* 0.28	
	Placebo group			**HDL-C (mg/dL)**	**HDL-C (mg/dL)**	
				Baseline 38.59 ± 1.68	Baseline 42.31 ± 2.22	0.18
				End 39.81 ± 1.89	End 41.57 ± 2.09	0.33
				*p =* 0.17	*p =* 0.38	
				**TC (mg/dL)**	**TC (mg/dL)**	
				Baseline 187.00 ± 7.48	Baseline 186.26 ± 7.8	0.94
				End 183.68 ± 7.06	End 190.57 ± 6.95	0.49
				*p =* 0.47	*p =* 0.51	
				**TG (mg/dL)**	**TG (mg/dL)**	
				Baseline 165.30 ± 41.20	Baseline 152.83 ± 21.12	0.20
				End 138.65 ± 41.70	End 164.89 ± 38.82	
				*p =* 0.03	*p =* 0.05	
				**LDL-C (mg/dL)**	**LDL-C (mg/dL)**	
				Baseline 126.96 ± 45.18	Baseline 117.70 ± 40.62	0.46
				End 116.60 ± 41.76	End 123.53 ± 23.76	
**Mazloomi et al., 2021** [89]	*Spirulina* sauce group;	2 g/day	8 weeks	*p =* 0.11	*p =* 0.71	
	Placebo group			**HDL-C (mg/dL)**	**HDL-C (mg/dL)**	
				Baseline 42.43 ± 8.29	Baseline 37.61 ± 11.27	0.10
				End 46.40 ± 11.64	End 38.58 ± 10.65	
				*p =* 0.02	*p =* 0.86	
				**TC (mg/dL)**	**TC (mg/dL)**	
				Baseline 202.48 ± 45	Baseline 185.96 ± 39.90	0.19
				End 186.75 ± 49.86	End 195.11 ± 24.86	
				*p =* 0.14	*p =* 0.42	
				**TG (g/L)**	**TG (g/L)**	
				Baseline 1.35 ± 0.4	Baseline 1.83 ± 0.82	0.11
				End 1.23 ± 0.57	End 1.97 ± 0.80	0.003
				NA	NA	
				**LDL-C (g/L)**	**LDL-C (g/L)**	
				Baseline 1.52 ± 0.33	Baseline 1.53 ± 0.36	0.88
				End 1.49 ± 0.33	End 1.43 ± 0.38	0.22
**Koite et al., 2022** [90]	*Spirulysat*^®^ group;	Arthrospira Liquid Extract	12 weeks	NA	NA	
	Placebo group	(Spirulysat^®^)		**HDL-C (g/L)**	**HDL-C (g/L)**	
				Baseline 0.54 ± 0.09	Baseline 0.47 ± 0.13	0.004
				End 0.55 ± 0.14	End 0.48 ± 0.18	0.031
				NA	NA	
				**TC (g/L)**	**TC (g/L)**	
				Baseline 2.33 ± 0.36	Baseline 2.36 ± 0.46	0.96
				End 2.30 ± 0.34	End 2.31 ± 0.43	0.57
				NA	NA	
				**TG (mmol/L)**	**TG (mmol/L)**	
				Baseline NA	Baseline NA	NA
				End 1.09 ± 0.63	End 1.06 ± 0.61	0.684
				NA	NA	
				**LDL-C (mmol/L)**	**LDL-C (mmol/L)**	
				Baseline NA	Baseline NA	NA
				End 2.75 ± 0.97	End 2.77 ± 1.07	0.677
**van den Driessche et al., 2020** [43]	*Spirulina* group;	4.5 g/day	17 days	NA	NA	
	Placebo group			**HDL-C (mmol/L)**	**HDL-C (mmol/L)**	
				Baseline NA	Baseline NA	NA
				End 1.52 ± 0.43	End 1.56 ± 0.49	0.273
				NA	NA	
				**TC (mmol/L)**	**TC (mmol/L)**	
				Baseline NA	Baseline NA	NA
				End 4.75 ± 1.00	End 4.81 ± 1.09	0.443
				NA	NA	

Abbreviations: TG, triglyceride; LDL, low-density lipoprotein; HDL, high-density lipoprotein; TC, total cholesterol; HIIT, high-intensity interval training; *Sp*, *Spirulina*; NA, not available.

## Data Availability

Not applicable.
